# A Decision Tree Model for Breast Reconstruction of Women with Breast Cancer: A Mixed Method Approach

**DOI:** 10.3390/ijerph18073579

**Published:** 2021-03-30

**Authors:** Eun Young Park, Myungsun Yi, Hye Sook Kim, Haejin Kim

**Affiliations:** 1 College of Nursing, Gachon University, Incheon 21936, Korea; parkeunyoung@gachon.ac.kr; 2 College of Nursing, Seoul National University, Seoul 03080, Korea; donam@snu.ac.kr; 3 Department of Nursing, Suwon Science College, Suwon 18516, Korea; hs_kim@ssc.ac.kr; 4 Department of Nursing, Suwon Women’s University, Suwon 16632, Korea

**Keywords:** breast cancer, decision-making, ethnography, mixed method design, breast reconstruction, decision tree model

## Abstract

The number of breast reconstructions following mastectomy has increased significantly during the last decades, but women are experiencing a number of conflicts with breast reconstruction decisions. The aim of this study was to develop a decision tree model of breast reconstruction and to examine its predictability. Mixed method design using ethnographic decision tree modeling was used. In the qualitative stage, data were collected using individual and focus group interviews and analyzed to construct a decision tree model. In the quantitative stage, the questionnaire was developed questions based on the criteria identified in the qualitative stage. A total of 61 women with breast cancer participated in 2017. Five major criteria: recovery of body image; impact on recurrence; recommendations from others; financial resources; and confirmation by physicians. The model also included nine predictive pathways. It turns out that the model predicted 90% of decisions concerning whether or not to have breast reconstruction. The findings indicate that the five criteria play a key role in decision-making about whether or not to have breast reconstruction. Thus, more comprehensive issues, including these five criteria, need to be integrated into an intervention for women with breast cancer to make their best decision on breast reconstruction.

## 1. Introduction

### 1.1. Background

Breast cancer is the most common cancer in women worldwide, with nearly 2.2 million women newly diagnosed in 2020, accounting for about 25 percent of all cancers in women [1]. At the same time, the ratio of early-stage breast cancer has risen mainly due to the early detection and diagnosis [2]. For example, in Korea, 62.4 percent of women with breast cancer (WBC) were at stage 0 or 1 at the diagnosis in 2018 [2]. And those with early-stage breast cancer tend to choose breast reconstruction (BR) as an option after breast resection. As a result, the number of BR following mastectomy has increased significantly during the last decades [3,4]. In the United States, for example, the ratio of BR has risen from 12% in 1998 to 36% in 2011 [4]. In Korea, the number of BR has increased from 1279 in 2014 to 5728 in 2017, showing about four times increase within three years [3].

BR has potential to improve body image for WBC with breast resection [5] and it does not necessarily delay detection of cancer recurrence or affect the outcomes of adjuvant chemotherapy [6,7]. No significant differences were found in treatment outcomes, such as local recurrence rate and survival rate, between WBC with BR and those with breast resection only [6,8]. BR also contributes to quality of life [5,9,10,11]. Other studies, however, have shown negative impacts of BR, such as regret [12,13,14] and decrease in overall satisfaction [12]. In terms of quality of life, some found no significant differences between WBC with BR and those with breast resection only [15] and others even reported lower quality of life in WBC with BR than those with breast resection only [13].

To improve positive outcomes and to minimize negative impacts from BR for WBC, a few intervention studies were conducted. However, some of these findings were discouraging [16]. To be effective, intervention needs to include not only cancer and treatment-related information, but also BR-related information, such as the type and size of the surgery area, duration of recovery, and risk of complications [13,17,18,19]. Since no differences are reported in local recurrence rates and treatment outcomes across types of reconstruction [8], greater consideration is required in selecting reconstruction type. In addition to these medical factors, other situations and conditions, such as psychological, social, and economic conditions need to be considered in decision-making [20,21]. Thus, more comprehensive understanding about meanings and perspectives about BR among WBC is needed to help them reach evidence-informed and value-congruent decisions about BR.

Several studies have identified factors that influenced the decision of BR. Factors that positively influenced BR decision included age, self-image, more clothing choices, the feeling of overcoming the cancer, functionality, bilateral mastectomy, access to private hospitals, quality and quantity of information, community or family support and early discussion of reconstructive options [22,23,24]. Reasons for avoiding BR were the fear of additional surgery, cancer recurrence and the belief that it was not important [22,24,25]. Previous studies have fragmentarily identified the factors that influenced the decision of BR using survey or interview methods. It is insufficient to understand what the main criterion that determines BR is and what the process is. This understanding can only be successfully achieved through patient-centered approaches into their decision- making process.

In particular, unlike other surgery, BR is a selective operation that is not essential. Thus, the decision and judgment of a woman with breast cancer is significant in considering all situations and conditions. Therefore, it is necessary to understand the specific process and criteria WBC utilize in choosing to undergo BR. This will provide significant education and counseling for WBC to make informed decisions.

### 1.2. Ethnographic Decision Tree Model

The purpose of this study was to understand how WBC decides whether or not to have BR from their perspectives. More specifically it aimed to identify major criteria influencing BR decisions and decisional pathways and to verify them, using ethnographic decision tree modeling. Ethnographic decision tree modeling is a mixed method design, consisting of qualitative and quantitative research methods, proposed by Gladwin [26]. It is based on the assumption that decision maker is an expert in making their decisions. According to Gladwin [26], the purpose of building a decision tree model is to present practical aspects of the decision situation. Models help simplify and understand specific situations. WBC who has to decide whether or not to have BR can objectively and clearly confirm her own situation by referring to the decision tree model.

In a qualitative study, decision tree model is constructed and then the constructed model is verified in a quantitative study. This method has various advantages. First, qualitative research helps attain a deep understanding about complicated decision-making situations and identify major theme or variables affecting decision-making. And verification of the results through quantitative research leads to more appropriate results by complementing limitations of qualitative method, such as a lack of generalization. It is also significantly advantageous as negative cases, those with different outcomes, can help in testing group models by classifying more relevant criteria [26,27].

Ethnographic decision tree modeling is a cyclical-discovery process of ethnographic research and context-sensitive models, which differentiates it from a rule-based model that processes data based on statistics [26]. Because the researcher uses ethnographic eliciting techniques to determine decision criteria, Ethnographic decision tree modeling has more realistic assumptions about an individual’s cognitive capabilities than do linear additive decision models. The situation in which WBC chooses BR is complexly influenced by various factors such as medical, physical, and emotional, psychosocial, and economic factors, so statistical decision methods may not be sufficient to reflect reality. In order to analyze and integrate the decision-making process from the perspective of the WBC, we tried to derive the decision-making process based on qualitative data. For this, research was conducted according to the model development processes suggested by Gladwin [26]. A team of researchers tried to derive the decision-making process closest to the WBC’s situation by performing data collection and model development according to the cyclical-discovery process.

## 2. Materials and Methods

### 2.1. Design and Process

**Figure 1:**
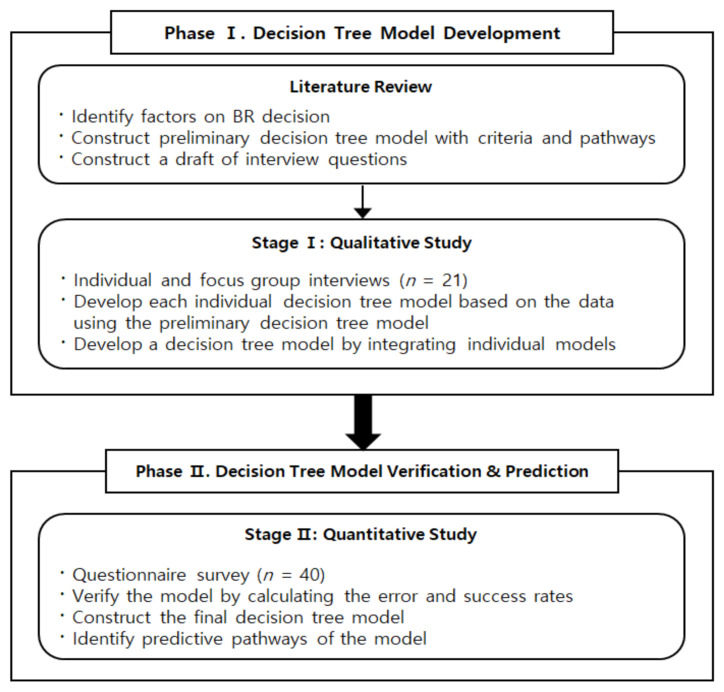
Research process using methodological triangulation.

This study utilized ethnographic decision tree modeling suggested by Gladwin [26] and Beck [27] to develop a decision tree model for BR of WBC, consisting of major criteria and pathways. The research process is shown in Figure 1. Before collecting data from the participants, we reviewed various qualitative and quantitative studies to identify factors influencing BR decisions and constructed a preliminary decision tree model with criteria and pathways. This preliminary model guided the model building in the qualitative research stage, which involves a series of ethnographic interview that is designed to describe actual BR decision-making experiences and diagram the choices of BR of the participants. Major criteria and pathways to decision-making were analyzed to construct a decision tree model by modifying the preliminary one.

In the quantitative research stage, a questionnaire was constructed using “yes” and “no” questions based on the decision-making criteria identified in the first stage. Data collected from the survey using the questionnaire was analyzed to predict the reliability of the model. The error rate and success rate were calculated from the criteria and pathways. If the model’s prediction rate reached 85–90%, the model was considered appropriate [27]. If the prediction rate was unsatisfactory, the decision-making criteria or pathways were reviewed until an appropriate prediction rate was obtained.

### 2.2. Participants

A total of 61 WBC participated in the study. The participants were recruited from several breast cancer patients’ associations in Korea. With support of the representatives of each association, the researchers attended the meeting or event to explain the purpose and methods of the study. After the researcher’s explanation, interviews or surveys were conducted with WBC who wishes to voluntarily participate in the study. Also, the interviews were carried out by adjusting the interview schedule to a convenient place and time for the participants. The participants were also recruited using a snowball sampling method [28]. Interviews were conducted with WBC by being introduced from those who participated in the interview or survey. The sample was intended to evenly include groups with and without BR, and groups with immediate reconstruction, with delayed reconstruction, with auto-transplantation, and with implant insertion.

In the qualitative research stage, 21 WBC participated to the interviews. Seventeen participants had BR and nine of them underwent BR immediately after mastectomy. Participants’ mean age was 53.95 years (41–66 years). In terms of the stage of diagnosis of breast cancer, six participants were in stage 0, eight in stage 1, and seven in stage 2, respectively. In the quantitative research stage, 40 WBC, including 20 with BR, participated to answer the questionnaires. Their mean age was 52.53 years (40–69 years), with 3 participants in stage 0, 7 in stage 1, 15 in stage 2, and 14 in stage 3, respectively. And one didn’t know her breast cancer stage. Among 20 participants with BR, ten underwent immediate reconstruction and the other halves had delayed reconstruction. Eight had autografts and 12 had artificial implant.

### 2.3. Data Collection

Qualitative data were collected in 2017 from 12 individual in-depth interviews and two focus group interviews with the participants. Semi-structured interview with open-ended questions was utilized to encourage the participants to recall and explain specific examples of decision-making and to maximize participants’ point of view. Semi-structured interview with open-ended questions was utilized to encourage the participants to recall and explain specific examples of decision-making and to maximize participants’ point of view. In the preliminary stage, previous studies on BR experiences were analyzed to identify the factors influencing BR decisions and interview questions were drawn [10,15,17,20,21]. As the interview progressed, we continued to ask more specific questions based on the participants’ interviews. In addition, the best alternatives that WBC can answer with yes and no were listed and used during the interviews to confirm if the preliminary model fit in actual situations and contexts. For example, ‘Was there a medical recommendation?’ was included to use during the interview. Interviews were conducted at each participant’s preferred time and place, and all were recorded and transcribed verbatim. Each interview lasted from 30 to 60 min, and follow-up interviews were done with several participants to verify what they said or meant. Participants were recruited until data was saturated.

Quantitative study was conducted for model verification, which aimed to determine the model’s ability to predict the decision-making of individuals in the same group. First, a model verification questionnaire consisting of yes/no questions was prepared for the decision-making criteria identified in stage 1, and quantitative data was collected from 40 WBC in 2017. The sample was intended to evenly include groups with and without BR, and groups with immediate reconstruction, with delayed reconstruction, with auto-transplantation, and with implant insertion.

### 2.4. Data Analysis

The specific research stages for constructing a model for the BR decision-making process followed the eight stages of ethnographic decision tree modeling proposed by Beck [27]:

(1) Identify decision-making issues to explore
(2) Specify a set of decision choices
(3) Master ethnographic interview skills: Researchers with experience in qualitative research were asked to participate in data collection and analysis.
(4) Participation observations should be included where possible, including data that manifest themselves as actions.
(5) WBC who underwent decision-making process for BR was selected to ensure the representativeness of the data. Sample numbers were collected until the data was saturated.
(6) Decision-making criteria were derived through inviting participants to recall and explain specific examples of decision-making. If an active objection was identified and confirmed, this was used as criteria.
(7) A decision-making tree was then developed by drawing flows and relationships between the derived criteria. To establish a clear statement stage with each individual decision-making tree and ensure each decision-making tree was appropriate, the researcher used the language and categories utilized by the participants. In developing individual models, group models were constructed by evaluating and modifying them in the following qualitative interviews.
(8) In the final stage, individual trees were combined to form a collective decision model that was revised several times in hierarchical order to ensure it is logical and predictable. The reason for establishing a decision-making tree was placed prior to the obstacle that hindered this choice or behaviour.

A decision making tree for each individual was developed by drawing flows and relationships between the derived criteria, and it was modified by evaluating in the following ethnographic interviews. If an active objection was identified and confirmed, it was used as a criterion. Individual tree models were then integrated to form a collective decision tree model. In this process, the reasons for choosing BR were placed prior to the obstacles that hindered these choices or behaviors. It was revised several times in hierarchical order to ensure it is logical and predictable.

In the stage of verification and prediction of the decision tree model, each participant followed the path predicted by the researchers and revised the model several times to check the error and calculate the error rate for each model. The success rate of the model was calculated with a percentage of the number of successful cases divided by the total number of cases. If the model success rate is more than 85%, the predictive power is said to be secured. Therefore, if the predictive power is within 85%, the model is modified by adding new criteria or reordering the paths of the model tree. In this process, we attempted to generalize decision-making criteria to eliminate repeatability and logically gather criteria. Care was taken to ensure that criteria categories were not too general to lose the cultural and ethnographic validity of the participants’ views. If the criteria were too broad, additional data was used to substantiate them into a meaningful category.

### 2.5. Ethical Considerations

The study was approved by the Research Ethics Review Committee (SNU 16-06-058) of the institution in charge of the researcher prior to conducting a research. The purpose of the study was explained to all participants before collecting data. The anonymity and confidentiality of the data were ensured. Participants were also informed that the participation can be withdrawn at any time during the study and that all data will only be used for research purposes. And written consent was obtained from all participants who voluntarily agreed to participate in the study. The contact information of the participants was collected for follow-up interviews, but the interviews were anonymous in the transcription and research results to prevent personal identification. Reimbursement was provided to all participants.

### 2.6. Rigor

The quality of this study was ensured by utilizing mixed method approach. In the qualitative research stage, credibility, fittingness, auditability, and confirmability proposed by Guba and Lincoln [29] were utilized to establish rigor of the study. To ensure credibility, open questions were used while bracketing the existing biases and stereotypes of the researcher. While data collection and analysis were performed simultaneously until the data became saturated. And the procedures of ethnographic decision tree modeling were followed carefully. Study team tried to maintain neutrality and exclude prejudice in the research process and results. In this study, the assumptions and prior understanding of the researcher were clarified in advance, and they were recognized throughout the entire research process, and efforts were made to take a neutral position from them. Credibility of data was established by interview recordings, verbatim transcriptions. Triangulation of the investigators and provision of several quotes from the participants in the findings also helped establish credibility of the analysis and interpretation. Auditability was ensured by providing exact procedures of this research process shown in Figure 1. In the quantitative study for model verification, the model secured over 85% predictive power. Researchers have experienced in nursing for cancer patient and conducting qualitative studies for WBC.

## 3. Results

### 3.1. Development of a Decision Tree Model

**Figure 2:**
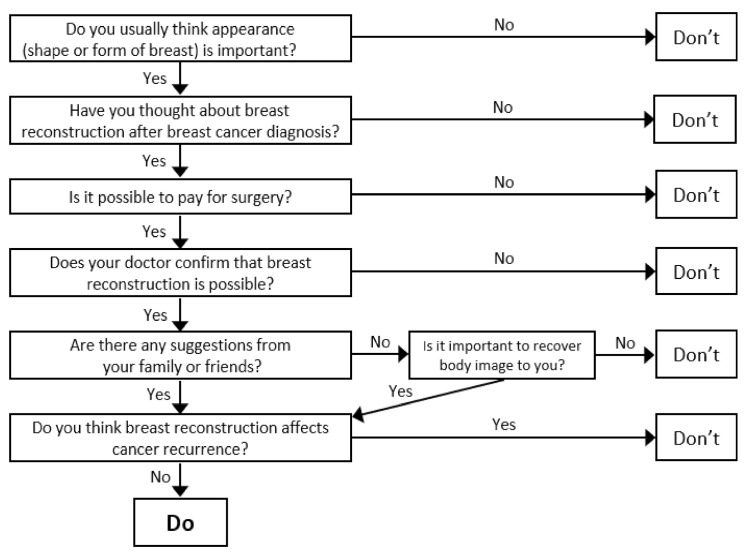
Modified preliminary decision tree model for breast reconstruction.

A preliminary decision tree model was developed with factors influencing BR decision making derived from the previous studies. Based on this preliminary model and analysis of the data from 17 participants who had BR, seven criteria affecting decision on BR were identified. They included “interest in physical appearance”, “considering BR”, “recovery of body image”, “impact on recurrence”, “recommendation from family and friends”, “financial resources” to pay for surgery, and “physician’s confirmation” that BR is possible. Data from four participants who did not undergo BR were also analyzed to identify the final decision-making criteria. Finally, modified preliminary decision tree model for BR was constructed as Figure 2. Based on these criteria, seven specific questions were then developed to verify the model in the ensuing portion of the study.

The first question in the decision tree was “Did you usually think your physical appearance (shape or form of breast) is important?” In response to the first question, the participants who answered “No” were placed in the rejection path of “BR”. This is because the difference between the participants who have undergone BR and those who did not depended on the importance of appearance. Only one participant did not think her appearance was not important and refused BR. If participants answered “Yes”, it led to questions 3–7.

Questions 3 to 5 are about external factors that influence the decision-making. In the BR decision, the participants considered whether the cost of surgery in addition to the costs of mastectomy and other cancer treatment was affordable. Participants also wanted advices on whether their medical and physical conditions were adequate for BR from their physicians in charge. They also sought additional advices on BR decisions from family and friends from a breast cancer support group.

After identifying external factors, participants were asked about themselves. Question 6: “Is it important to recover body image to you?”. This was an important criterion for BR decision because loss of breast and gender identity was regarded as a severe impairment rather than a simple physical health issue.

Question 7: “Do you think BR affects cancer recurrence?” This question asks about subjective feeling about or worry that BR might affect treatment or recurrence of cancer. Although all other criteria were positive for BR, the participants who answered “Yes” in this question did not ultimately choose to have BR.

The sequence of the decision-making criteria was not fixed in the model as it depends on each participant’s situation. Moreover, as BR can be conducted simultaneously with mastectomy or as a separate operation over time, the decision-making cycle can be resumed even if the final stage of the decision-making process has been reached. Thus, the criteria could work in a recurrent way.

### 3.2. Verification and Prediction of the Decision Tree Model

**Figure 3:**
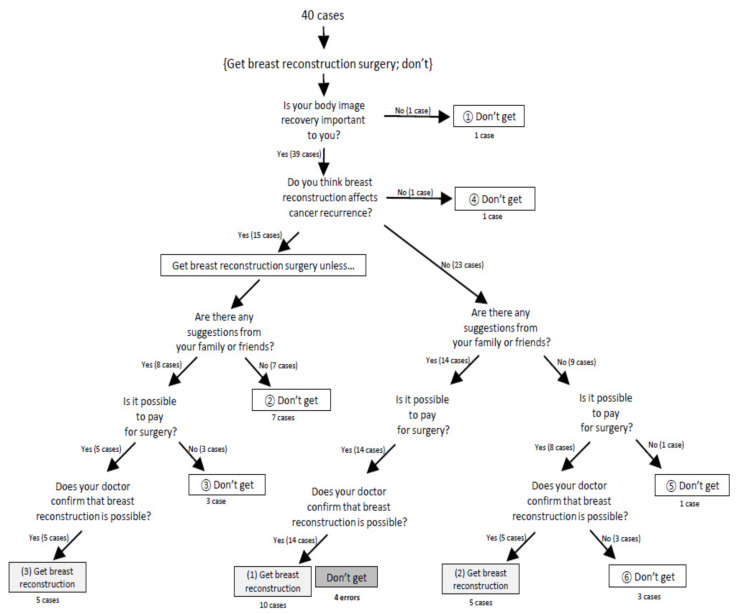
Final decision tree model for breast reconstruction.

A model verification questionnaire was constructed as “yes/no” questions for the decision-making criteria identified in the first stage. It included seven questions, such as ‘Did you usually think appearance (shape of form of breast) is important?’ and ‘Do you think BR affects cancer recurrence?’ The survey was conducted on 20 WBC who had BR after mastectomy and 20 who did not undergo BR. Based on the survey results, the final model for decision-making for BR was constructed as Figure 3. In this process, questions 1 and 2 were removed as they were considered repetitious and unpredictable. The predictive power of the final model was found to be 90%. In cases of women who underwent BR, 100% of them followed this model. In cases of 20 WBC without BR, four participants did not follow this model.

- Predictive pathways to decide to have BR

There were three predictive pathways to decide to have BR. Pathway 1 to decide to have BR was comparatively confirmed without any resistance by those who value the recovery of the body. There were also positive answers about possible recurrence of cancer caused by BR and supporting of family or friends, as well as the ability to pay for the surgery and doctor’s confirmation (10 cases). Pathway 2 was related to WBC who did not receive support from others. Even without support from family or friends, they decided to BR when their economic ability and doctor’ confirmation were satisfied (5 cases). Pathway 3 was drawn from WBC worried about a relapse. Despite concerns about recurrence, if the answers to other questions were ‘Yes’, it was revealed that they finally chose BR (5 cases).

Two major characteristics in women who had decided to have BR were identified. First, the participants regarded recovery of body image as important as breast cancer treatment. All participants who had BR answered “Yes” to the question “Was your body image recovery important to you?” Especially for those who received only mastectomy without reconstruction in the initial treatment decision, recovery of body image was major decision criteria to decide to undergo “delayed BR”.

*I had lived without breast about two years. But, you know, I completely lost my self-esteem. I was so depressed that I even thought about suicide. Finally, it came to my mind that I will live as a woman with confidence even if I can live only one day!*

(Participant 15)

*Without breast, I feel like she is not a woman. So I thought that others would think that I am not a woman when they saw me. Honestly, men touch women’s breast. As a wife, I wanted to be a woman to my husband. It seemed to be my pride…*

(Participant 12)

The second characteristic was that they received active support from family, friends or other WBC. Seventy-five percent of the participants who had BR reported receiving support. In particular, among the 15 participants who perceived that BR affects cancer recurrence, five participants who received support from people had undergone BR.

*One day, about two years have passed after mastectomy, I looked at my body and I suddenly realized that I needed to have BR. So, I talked about it in front of my family. And my husband said, “You can have it any time you want! You could have done it from the beginning, but you didn’t do it!” And my daughter said, “Mom, you do it! You know it’s great”. Anyway, I couldn’t decide at that time. But, later on, I just choose to do it as I was being pushed away by my family*.

(Participant 14)

*When I was in hospital for cancer treatment, other patient recommended breast reconstruction… very strongly. “I’m so happy and satisfied with the surgery!” She didn’t say that surgery was difficult at all… she only talked about the good points*

(Participant 13)

*My family thought that my body would be twisted without a breast. “If you don’t have surgery, your body will bend. Are you okay?” My daughter persuaded me to do breast reconstruction by saying so*.

(Participant 17)

- Predictive pathways to decide against to have BR

Six predictive pathways were identified to decide against to have BR. Pathway 1 was in case that woman who didn’t care about the body image (1 case). Pathway 2, 3 were related to WBC who emphasized a recovery of body image but concerned about cancer recurrence caused by BR. Final decisions of them were made not to have reconstruction in cases any support from friends or family (seven cases), or in cases surgery costs were burdensome even with support from others (three cases). Pathway 4–6 were related to WBC thought that surgery did not affect cancer recurrence, and they did not receive reconstruction in the end because of the burden of surgery cost or absence of confirmation from the doctor (five cases).

Women who chose not to undergo BR generally had two characteristics. First, they did not have active recommendations from family, friends or other patients. Seven women answered that appearance was important to them, but they could not choose BR because they did not receive support from others. Therefore, an important factor is the question “Are there any recommendations from your family or friends?” In particular, the negative experiences of other women with BR had a significant effect on the decision-making. One participant addressed this issue as follows:

*Another friend [who had breast reconstruction] told me that she finished her cancer treatment and when she had to decide to have surgery [BR] again, it was very hard in terms of time, effort, and cost she put. And, she said she wouldn’t do it again anymore. When I heard about that, I also didn’t want to have that kind of surgery because I thought ‘what should I do if I would have the same feeling and results like her?’*

(Participant 18)

The second characteristic is about concern whether WBC was financially able to pay the cost of BR. Whilst the participants hoped that BR will increase self-esteem and reduce social constraints, the financial burden of BR became an unacceptable factor even with family support for BR.

*The insurance didn’t cover it at that time, and moreover I had to pay VAT on the surgery fees. It was so unfair that I was sick and cost a lot of money. I’m sorry to have had a mastectomy… Should I undergo breast reconstruction while paying the VAT of the surgery… That’s too sad and I am very angry*.

(Participant 14)

## 4. Discussion

This study aimed to develop a decision tree model for BR after mastectomy among WBC. As a result, a decision tree model that includes five major factors affecting the decision whether or not to have a BR was developed. Major factors include recovery of body image; impact on recurrence; recommendation from others; financial resources; and confirmation by physicians. The decision tree model also provides three predictive pathways to deciding to have a BR and six predictive pathways to deciding against it. The decision tree model predicted 90% of decisions whether or not to have a BR. Although this data is in 2017, breast cancer is still the most prevalent cancer among Korean women and patients are in a situation where they have to choose BR or not. To date, research showing the decision-making process of WBC is insufficient. So this finding is meaningful to understand WBC’s decision-making process and to suggest further research directions.

The study identified the recovery of body image as the most important factor in decision to have BR for WBC. This is consistent with numerous previous findings [5,20,21,30]. This is also related to the findings that fear of losing femininity due to mastectomy affected the decisions to have BR [17,31,32]. Indeed, femininity including body image is important for most women. Especially in the appearance-oriented society like Korea, the perceptions of others are very important, and as a result, physical damage can be as great as breast cancer [21]. For example, in the study of hysterectomy decision-tree model in Taiwan [33] the participants would reject surgery to preserve their uterus. Thus, oncologists must be aware that female organs have a greater significance than other parts of the body for women. Recovery of body image and femininity must be assessed in decision whether to have BR or not. However, it is important to emphasize that body image recovery is essential not only for their appearance but also for their proper posture and physical health.

This study found that women’ perceptions about whether BR affected cancer recurrence was an important factor in deciding on BR. If women think BR may affect breast cancer recurrence or delay in finding a recurrence, BR decisions may be delayed or abandoned. This study found that WBC who underwent BR was more confident than the others on this issue. However, some WBC underwent BR despite the perception that BR may affect recurrence. As relapse is the greatest health risk for cancer patients and the most important concern of the cancer patients in general [34], false perceptions and fears about cancer recurrence can interfere with effective decision-making or cause anxiety even after deciding to undergo BR. Thus, WBC must be updated to make informed decisions about their BR without any misunderstanding of BR surgery.

This study found that WBC depended a lot on recommendations or opinions of surrounding people in the decision-making process for BR. This was also confirmed by Fasse et al. [35] who studied the decision-making process in couples. Cancer patients in general are influenced by the opinions of their social groups including family and friends [36]. In this study, the support of husband and/or children was important, but the influence of other WBC through the breast cancer patients’ association was also significant. In particular, the experiences of WBC who had already undergone BR had a significant effect on the WBC considering BR. This effect confirms the findings that support groups play a key role in hysterectomy decision-making since they provide relevant information as well as emotional support [33]. Therefore, oncology professionals need to inform the patients’ families with enough information on BR decisions, and they need to include information from support groups to reach evidence-informed decisions.

The study results also showed that BR decisions were affected by the patient’s financial resources, consistent with many findings that household income or having private insurance affects the rate of BR [20,37,38,39]. However, as the number of health care beneficiaries increase, such as in Korea, the decision-making criteria for BR must be reviewed for any changes.

The physicians’ positive feedback on BR was an additional major factor in the final stage of decision-making. In this study, participants wanted to obtain verbal confirmation from their physicians to see if they were encouraged to have BR. If the physician’s response was vague or negative, the participants gave up BR. In previous studies, cancer patients considered the medical staff’s instructions to be the most authoritative, rather than the information obtained from their surroundings [40] which are similar to the report that absolute confidence in physicians is a factor in deciding for BR [21]. Physician’s consultation with a WBC about BR also affects the treatment decisions [41] and if a physician provides WBC with additional information about treatment and emotional support, this can alleviate the conflict and anxiety in the decision-making process. This suggests that the role of oncology nurses as well as physicians needs to be reinforced to provide appropriate information and counseling for WBC who consider BR to reduce decision conflict.

Previous studies found that the size of surgery, length of recovery, and risks of complications were important in deciding for BR and the main reason for not undergoing BR was the fear of complications [17,20]. Unlike previous studies, however, concerns about BR surgery or its complications were not identified as major criteria for decision-making in this study. While some participants were concerned about the length of time and the complications associated with BR when choosing the type of BR, this was not a major factor influencing decision-making. This phenomenon may have resulted from the fact that not enough information on side effects or complications of BR was known among oncologists and WBC in Korea. In addition, there may be a difference in the degree of participation in BR decision-making depending on the patient’s decision-making style [42]. Therefore, future studies are needed to determine whether the decision-making criteria or the pathways in the model change according to the patient’s decision-making style.

In summary, the study showed that BR decision is not easy for most WBC, requiring significant psychosocial energy over a long time. This study, in particular, found that recovery of body image, recurrence considerations, support of family and other WBC, financial resources, and physicians’ confirmation as key criteria in the process of BR decisions. With the exception of some participants, most participants’ decision-making derived from the decision tree model with 90% of predictive power. The results of the study suggest that oncology professionals need to pay attention to these five important criteria when providing education and counseling to help WBC make their best decision on BR.

There are many models that support decision-making. Multi-criteria decision-making (MCDM), a representative decision-making model applied to decisions for treatment, is a process of determining the best alternatives or it’s rankings by measuring and integrating the preferences of a number of alternatives that are considered as an option under each criteria [43]. The analytic hierarchy process (AHP) and technique for order of preference by similarity to ideal solution (TOPSIS) are examples of MCDM methods used in the medical field [43,44]. And the characteristic objects method (COMET) is presented as a potential MCDM for use in medical issues [45,46]. In particular, COMET has the advantage of no reversal of ranks, so it is being proposed as a new method for solving medical problems [45,46]. In addition to the ethnographic decision tree modeling, the comparison with results of applying other research methods is considered meaningful. In order to apply MCDM method, weights of criteria must be identified or fuzzy numbers must be defined. BR is not a treatment for the cure, but a selective treatment for the recovery of body images. So, emotional and psychosocial factors such as the individual’s beliefs, advice from a significant person, cultural consciousness of the community about health and body are more meaningful in decision of BR than cure rate or survival rate. By quantifying the WBC’s subjective factors, it is possible to more objectively measure BR decision making in a fuzzy environment not in an uncertain environment that contains a lot of subjectivity [44]. In addition, the relative importance of each factor influencing BR decision-making can be identified and analyzed with AHP. These efforts will help to improve the ambiguity and inaccuracies of the result of this study.

This study has some limitations to be considered while interpreting the results. First, the research subjects were recruited by convenience sampling, so the research results cannot be generalized to WBCs around the world. Since culture is one of the important factors that influence on how people think, behave, and decide, application of the findings is limited to WBC living in cultures that are similar to Korea. Korea is considered a collectivistic society that emphasizes group harmony in interpersonal relationships, while individualistic society advocates interests of the individual over a group as a whole [47]. Thus, the findings would reflect more interdependent characteristics of the participants’ interactions while making decisions about BR. And application of the findings might also be limited to WBC living in health care environments that are similar to Korea. So we suggest a repetitive study with the subjects recruited from different geographical areas and with larger samples will determine whether the decision-making tree model could be applied to all women considering BR. Second, the data collection interview started for a long time. Therefore, we propose repeated studies for WBC, which determines recent BR. Third, the accuracy of data collected in this study depends on the women with breast cancer, which might cause errors in data especially time interval from diagnosis of cancer to breast reconstruction. We suggest another study with women awaiting a BR decision to remove error occurred due to memory loss.

In spite of these limitations, this study could provide the underlying data to support medical staffs counselling WBC and strengthen the decision-making authority of WBC. Moreover, this study could offer the fundamental data to enhancing the competence of nurses in cultural nursing in WBC from various cultural backgrounds and contribute to the development of cultural nursing theory expansion, so the findings suggest that it is necessary to develop education guidelines for medical staff or interventions for WBC based on the decision tree model.

## 5. Conclusions

The number of BR after mastectomy in WBC has increased, but not enough studies about BR decisions among WBC were conducted to support effective decision-making. This mixed method study provides a decision tree model for BR of WBC by identifying major factors and pathways and providing experiences related to these areas. These results of the study would support oncology professionals collaboratively help WBC to reach evidence-informed and value-congruent decisions about BR. In particular, it demonstrates that the WBC’s BR decision-making should consider cultural factors. It is highly contributing to the increase in cultural nursing capabilities of oncology nurses. The results of this study contribute to promoting understanding of the decision-making process of treatment for vulnerable groups such as women and the elderly. In addition, the phenomenon that culture contributes to health-related decision-making was presented as the basis of cultural nursing by the ethnographic research contributed to the field of nursing research.

## Data Availability

The data that support the findings of this study are available from the corresponding author, upon reasonable request.
